# Sleep quality is a predictor of muscle mass, strength, quality of life, anxiety and depression in older adults with obesity

**DOI:** 10.1038/s41598-023-37921-4

**Published:** 2023-07-12

**Authors:** Rafael Genario, Saulo Gil, Gersiel Oliveira-Júnior, Alice Erwig Leitão, Tathiane Franco, Ruan Célio dos Santos Sales, Eduardo Ferriolli, Alexandre Leopold Busse, Wilson Jacob Filho, Bruno Gualano, Hamilton Roschel

**Affiliations:** 1 Applied Physiology and Nutrition Research Group, School of Physical Education and Sport, School of Medicine, Universidade de Sao Paulo, Sao Paulo, Brazil; 2 Rheumatology Division, Clinical Hospital, Hospital das Clinicas HCFMUSP, Faculdade de Medicina, Universidade de Sao Paulo, Sao Paulo, Brazil; 3 Division of Internal and Geriatric Medicine, Department of Internal Medicine-Ribeirão Preto Medical School, Universidade de São Paulo, Ribeirão Preto, SP Brazil; 4 Laboratorio de Investigacao Medica em Envelhecimento (LIM-66), Serviço de Geriatria, Hospital das Clinicas HCFMUSP, Faculdade de Medicina, Universidade de São Paulo, São Paulo, SP Brazil

**Keywords:** Metabolism, Physiology, Psychology

## Abstract

We aimed to investigate associations between sleep quality with selected quantitative and qualitative parameters of health in older individuals with obesity. Cross-sectional assessment (n = 95 men/women; ≥ 65 years; BMI ≥ 30 kg/m^2^) of sleep quality, body composition, handgrip strength, quality-of-life, anxiety/depression. Mean PSQI score was 6.3. Poor sleepers (n = 49) presented lower appendicular lean mass (ALM) (16.2 vs 17.8 kg; *p* = 0.0273), ALM/BMI (0.47 vs 0.53 kg/BMI; *p* = 0.0085), fat mass (48.6 vs 46.6%; *p* = 0.0464), handgrip strength (19.7 vs 22.0 kgf; *p* = 0.0542) and handgrip/BMI (0.57 vs 0.66 kgf/BMI; *p* = 0.0242) than good sleepers. They also had higher anxiety (8.6 vs 5.6; *p* = 0.0100) and depression (4.8 vs 3.2; *p* = 0.0197) scores, worse health-related quality-of-life and lower scores in mental (62.8 vs 73.0; *p* = 0.0223) and physical (52.9 vs 67.3; *p* = 0.0015) domains. Adjusted models showed that PSQI was negatively associated with ALM (β = − 0.13, 95% CI − 0.25; − 0.01) and health-related quality of life on physical (β = − 2.76, 95% CI − 3.82; − 1.70) and mental (β = − 2.25, 95% CI − 3.38; − 1.12) domains, and positively associated with anxiety (β = 0.57; 95% CI 0.26; 0.87) and depression (β = 0.31; 95% CI 0.13; 0.49). Poor sleep quality associates with impaired selected quantitative and qualitative parameters of health. Additionally, sleep quality was shown as an independent predictor of ALM, health-related quality-of-life, anxiety and depression in older individuals with obesity.

## Introduction

Obesity in older population is an increasing issue worldwide^1^. The presence of obesity during aging may impact distinct physiological process (e.g., anabolic response and glucose metabolism)^2–4^ and, as a consequence, physical and mental health^5,6^. Additionally, obesity may exacerbate the inherent adverse effects of aging on sleep disorders^7,8^.

Sleep is recognized as a critical determinant of health and well-being across distinct populations^9,10^. Sleep impairments are common in older individuals, and it has been linked to physical disability, among other health outcomes^11–14^. Indeed, previous data have also shown sleep deprivation to induce anabolic resistance and a pro-catabolic environment^15^. It is noteworthy that the adverse effects of poor sleep quality may exacerbate the anabolic resistance commonly observed in older adults, especially in those presenting with obesity^3^, which may further contribute to disturbances in body composition (e.g., high fat mass and low lean mass), muscle weakness, and poor quality of life. As sleep disturbances may superimpose on obesity in respect of its detrimental effects, it is reasonable to speculate that poor sleep quality may be associated with lower lean mass, physical disability, and poor quality of life in older individuals with obesity, a population that has been poorly investigated.

Therefore, the aim of this study was to investigate the possible associations between sleep quality and on body composition, muscle strength, anxiety, depression, and health-related quality of life in older individuals with obesity.

## Results

Ninety-five older participants were evaluated (good sleepers: n = 46; poor sleepers: n = 49). Good sleepers showed lower global PSQI score (3.54 ± 1.29 vs. 8.86 ± 3.22; 95% CI − 6.326 to − 4.301;* p* ≤ 0.001) than poor sleepers. Table 1 details demographic and clinical characteristics for each group.

**Table 1:** Demographic and clinical characteristics.

Baseline characteristics	Good sleepers (n = 46)	Poor sleepers (n = 49)
Sex (*n*, %)		
Female	36 (78%)	43 (88%)
Male	10 (22%)	6 (12%)
Age (years) (mean ± SD)	72.43 ± 6.12	73.71 ± 6.22
Sleep (mean ± SD)		
Global PSQI score	3.54 ± 1.29	8.86 ± 3.22
Body composition (mean ± SD)		
Body weight (kg)	84.35 ± 13.57	83.35 ± 12.24
Height (m)	1.58 ± 0.08	1.55 ± 0.08
BMI (kg/m^2^)	33.73 ± 3.89	34.74 ± 3.91
Fat mass (Kg)	38.83 ± 8.47	39.72 ± 8.20
Fat mass (%)	46.59 ± 4.84	48.59 ± 4.77
ALM (kg)	17.79 ± 3.58	16.24 ± 3.11
ALM/BMI (kg/BMI)	0.53 ± 0.10	0.47 ± 0.10
Muscle strength (mean ± SD)		
Handgrip (kg)	22.00 ± 6.18	19.73 ± 5.12
Handgrip/BMI (kg)	0.66 ± 0.20	0.57 ± 0.16
Quality of life (mean ± SD)		
SF-36 (mental health)	73.05 ± 19.75	62.83 ± 21.56
SF-36 (physical health)	67.26 ± 16.68	52.93 ± 23.58
Comorbidities (*n*, %)		
Diabetes	9 (19.6%)	8 (16.3%)
Hypertension	34 (73.9%)	24 (48.9%)
Pulmonary diseases	1 (2%)	2 (4.1%)
Psychiatric diseases	3 (6.5%)	5 (10.2%)
Rheumatic diseases	9 (19.6%)	10 (20.4%)
Use of drugs (*n*, %)		
Use of anxiolytics	2 (4.3%)	5 (10.2%)
Use of antidepressant	6 (13.0%)	9 (18.4%)

*ALM* appendicular lean mass, *BMI* body mass index, *KgF* kilogram-force, *PSQI* Pittsburgh sleep quality index, *SD* standard deviation, *SF-36* Short Form Health Survey.

**Figure 1:**
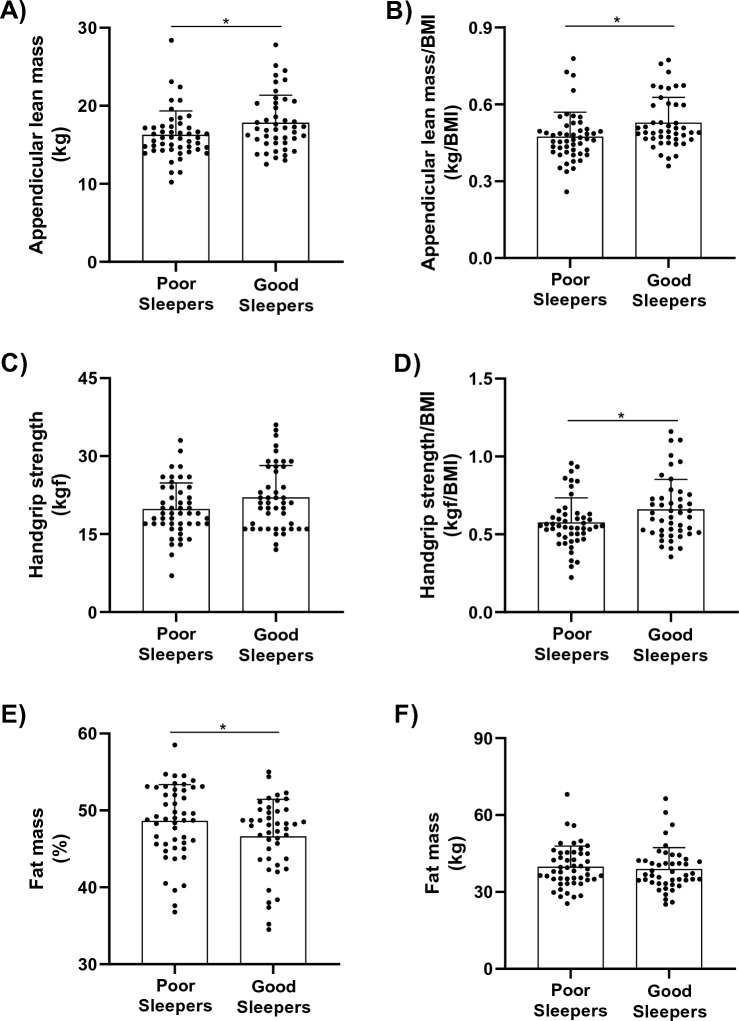
Appendicular lean mass, fat mass and handgrip strength in poor (n = 49) and good sleepers (n = 46) patients. (**A**) Appendicular lean mass; (**B**) appendicular lean mass adjusted by BMI; (**C**) fat mass; (**D**) percentage of fat mass; (**E**) handgrip strength; (**F**) handgrip strength adjusted by BMI. *BMI* body mass index. Data is presented as individual data, mean, and standard deviation. *Indicates P < 0.05 for between-group comparisons using independent t-test.

Poor sleepers showed lower values of ALM (16.2 ± 3.1 vs. 17.8 ± 3.6 kg; 95% CI 0.17–2.93*; p* = 0.0273), ALM/BMI (0.47 ± 0.10 vs. 0.53 ± 0.10 kg/BMI; 95% CI 0.01–0.09*; p* = 0.0085) and handgrip/BMI (0.57 ± 0.16 vs. 0.66 ± 0.20 kgf/BMI; 95% CI 0.01–0.16; *p* = 0.0242) than good sleepers. In addition, poor sleepers demonstrated a higher relative (%) (48.6 ± 8.2 vs. 46.6 ± 8.5%; 95% CI − 3.97, − 0.03; *p* = 0.0464) but not absolute (kg) (*p* > 0.05) fat mass in comparison with good sleepers (Fig. 1, Panels A-F).

**Figure 2:**
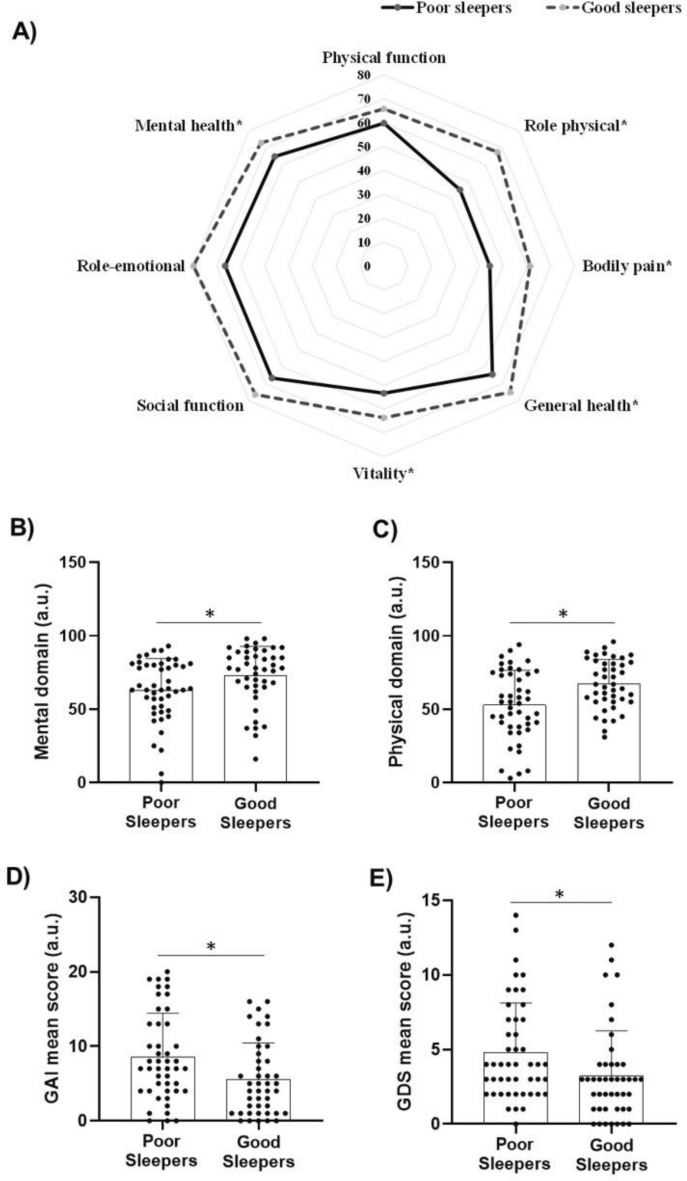
Healthy-related quality of life, anxiety (GAI), and depression (GDI) scores in poor (n = 49) and good sleepers (n = 46) patients. (**A**) Short Form-36 health survey in each domain; (**B**) mental health domain; (**C**) physical health domain; (**D**) geriatric anxiety inventory; (**E**) geriatric depression scale; *a.u.* arbitrary units. Data is presented as individual data, mean, and standard deviation. *Indicates P < 0.05 for between-group comparisons using independent t-test.

The SF-36 analyses revealed that poor sleepers had lower values in mental (62.8 ± 21.6 vs. 73.0 ± 19.7; 95% CI 1.52–18.92; *p* = 0.0223) and physical (52.9 ± 23.6 vs. 67.3 ± 16.7; 95% CI 5.75–22.89; *p* = 0.0015) domains compared with good sleepers. As for SF-36 sub-scales, poor sleepers showed lower scores in the role-physical (45.1 ± 41.0 vs. 67.4 ± 36.4; 95% CI 6.01–38.65; *p* = 0.007), general health (64.3 ± 27.1 vs. 74.9 ± 16.2; 95% CI 1.25–19.96; *p* = 0.026), vitality (53.4 ± 21.4 vs. 63.7 ± 21.0; 95% CI 1.41–19.30; *p* = 0.023), mental health (64.8 ± 21.2 vs. 72.9 ± 17.6; 95% CI − 0.05 to 16.35; *p* = 0.051), and bodily pain (44.4 ± 22.8 vs. 61.3 ± 22.9; 95% CI 7.15–26.64; *p* ≤ 0.001) in comparison with good sleepers. No between-group differences were observed for role-emotional and physical and social function (all *p* > 0.05) (Fig. 2, Panels A-C).

Poorer sleepers exhibited higher scores of both anxiety (8.59 ± 5.85 vs. 5.59 ± 4.88; 95% CI − 5.26 to − 0.74; *p* = 0.010) and depression (4.81 ± 3.31 vs. 3.23 ± 3.03; 95% CI − 2.90 to − 0.26; *p* = 0.019) when compared with good sleepers (Fig. 2, Panels D-E).

Crude linear regression model showed an inverse association between sleep quality score (the higher the score, the worse the sleep quality) and ALM/BMI (β = −0.01; 95% CI − 0.01 to 0.01; *p* = 0.080), health-related quality of life for both physical (β = −2.75; 95% CI − 3.87 to − 1.64; *p* ≤ 0.001) and mental domains (β = −2.37; 95% CI − 3.50 to − 1.25; *p* ≤ 0.001), and anxiety (β = 0.60; 95% CI 0.31–0.90; *p* ≤ 0.001) and depression (β = 0.32; 95% CI 0.15–0.50; *p* ≤ 0.001) scores. These associations remained statistically significant after adjusted for covariates [Model 1: ALM/BMI (β = −0.01; 95% CI − 0.02 to − 0.01; *p* = 0.035), physical (β = −2.76; 95% CI − 3.85 to − 1.66; *p* ≤ 0.001) and mental (β = −2.37; 95% CI − 3.51 to − 1.27; *p* ≤ 0.001) domains, Anxiety (β = 0.32; 95% CI 0.31–0.90; *p* < 0.001), and Depression (β = 0.32; 95% CI 0.15–0.50; *p* < 0.001); Model 2: ALM (β = −0.13; 95% CI − 0.25 to − 0.01; p = 0.036); physical (β = −2.76; 95% CI − 3.82 to − 1.70; *p* ≤ 0.001) and mental (β = −2.25; 95% CI − 3.38 to − 1.12; *p* ≤ 0.001) domains, and Anxiety (β = 0.57; 95% CI 0.26–0.87; *p* < 0.001), and Depression (β = 0.31; 95% CI 0.13–0.49; *p* < 0.001)] (Table 2). No statistically significant association was detected between sleep quality scores and absolute and relative handgrip strength (both *p* > 0.05). Quality of regression models (i.e., Q-Q plot, Cook’s distance, R^2^, root mean square error and variance inflation factor) can be checked in Supplementary Figs. S1–S8 and Supplementary Tables S1 and S2.

**Table 2:** Linear regression analyses of the association between sleep quality with appendicular lean mass, handgrip strength and quality of life outcomes.

Outcome	Crude model			Model 1^a^			Model 2^b^		
	β	95% IC	*p* value	β	95% IC	*p* value	β	95% IC	*p* value
ALM	− 0.14	− 0.33 to 0.06	0.164	− 0.10	− 0.23 to 0.04	0.153	− 0.13	− 0.25 to − 0.01	**0.03**
ALM/BMI	− 0.01	− 0.01 to 0.01	0.080	− 0.01	− 0.02 to − 0.01	**0.035**	− 0.01	− 0.02 to 0.01	0.05
Handgrip strength	− 0.23	− 0.55 to 0.10	0.167	− 0.17	− 0.43 to 0.09	0.192	− 0.20	− 0.46 to 0.07	0.140
Handgrip strength/BMI	− 0.01	− 0.02 to 0.01	0.101	− 0.01	− 0.01 to 0.01	0.141	− 0.01	− 0.01 to 0.01	0.159
SF-36—physical domain	− 2.75	− 3.87 to − 1.64	** < 0.001**	− 2.76	− 3.85 to − 1.66	** < 0.001**	− 2.76	− 3.82 to − 1.70	** < 0.001**
SF-36—mental domain	− 2.37	− 3.50 to − 1.25	** < 0.001**	− 2.39	− 3.51 to − 1.27	** < 0.001**	− 2.25	− 3.38 to − 1.12	** < 0.001**
Geriatric anxiety inventory	0.60	0.31 to 0.90	** < 0.001**	0.32	0.31 to 0.90	** < 0.001**	0.57	0.26 to 0.87	** < 0.001**
Geriatric depression scale	0.32	0.15 to 0.50	** < 0.001**	0.32	0.15 to 0.50	** < 0.001**	0.31	0.13 to 0.49	** < 0.001**

*ALM* appendicular lean mass, *BMI* body mass index, *SF-36* short form (36) health survey.

Significant values are given in bold.

^a^Linear regression models were adjusted by age (as continuous variable) and sex (male or female).

^b^Linear regression models were adjusted by age (as continuous variable), sex (male or female), body mass index (as continuous variable), type II diabetes (yes or no), pulmonary diseases (yes or no), psychiatric diseases (yes or no), hypertension (yes or no) and rheumatic disease (yes or no).

## Discussion

In the current study we investigated the impact of sleep quality on body composition, muscle strength, anxiety and depression, and health-related quality of life in older individuals with obesity. Our main findings indicate that older individuals with obesity classified as poor sleepers exhibited lower absolute and relative appendicular lean mass, lower values of handgrip strength, and higher percentage of body fat when compared with good sleepers. Also, we observed worse scores of mental and physical health, anxiety, and depression in poor vs. good sleepers. Finally, adjusted linear regression models revealed that poor sleep quality is associated with lower appendicular lean mass and poorer scores of mental and physical health, and greater scores of anxiety and depression.

Acute and chronic sleep loss are widely recognized to negatively influence physiological outcomes^16^. For instance, impaired sleep reduces the expression of anabolic hormones (e.g., GH, IGF-1 and testosterone), in addition to increasing the production of molecules with catabolic potential (e.g., myostatin and glucocorticoid hormones), promoting a scenario that can impair muscle protein synthesis (MPS)^17,18^. In fact, a recent study demonstrated that a single night of sleep deprivation is sufficient to promote an 18% reduction in MPS, indicating mechanistic precursors driving the metabolic dysfunction and body composition changes such as reduced lean body mass^15^. These results are further corroborated by the demonstration that a 5-day sleep restriction (4 h in bed/night) reduced MPS by ~ 19%^18^. Finally, a 3-h sleep reduction from habitual total sleep time resulted in greater loss of lean mass during moderate caloric restriction in overweight adults when compared to calorie restriction alone^19^.

Importantly, obesity may exacerbate anabolic resistance, thus negatively impacting muscle mass and strength of older individuals with obesity^2,3,20^. Our findings indicate that the coexistence of obesity and poor sleep quality potentiate these negative effects on muscle mass and strength in older people. These findings are of clinical relevance, as aging itself is thought to negatively impact body composition, and, hence, cardiometabolic health, which may be further impaired by sleep disturbances.

Obesity may also negatively impact psychological health, including structural changes in the brain^21,22^ and increased risk for psychiatric diseases^23,24^. Similarly, psychiatric disorders such as anxiety and depression are highly prevalent in older individuals^25^, which may be exacerbated by the presence of sleep disturbances^26,27^, with important implications in quality of life. Our findings support these data by showing worse scores in anxiety, depression, and quality of life in poor sleepers, suggesting that sleep quality may increase the risk of psychiatric disorders and impairments in quality of life in older individuals with obesity.

In the current study, regression analyses revealed that sleep quality is a significant predictor of ALM, health-related quality of life (physical and mental domains), anxiety and depression regardless of age, sex, body mass index, type II diabetes, pulmonary diseases, psychiatric diseases, hypertension, and rheumatic disease. These findings reinforce the potential role of sleep quality on general health status of older individuals with obesity. As this is an already at-risk population, it appears that sleep quality should be targeted to avoid further deterioration on overall health.

It is noteworthy that sleep represents a set of many physiologic processes under primarily neurobiological regulation that impacts many physiologic systems^9^. Therefore, it is recognized as a critical determinant of health^9,10^. Sleep quality may be affected by both internal (e.g., chronic pain, stress, mental health issues, snoring, and sleep disorders) and external factors (e.g., blue light, jet lag, medications, sleep environment, sleep schedule, caffeine and alcohol intake, physical activity and certain foods)^28–35^. In this scenario, it is reasonable to assume that poor sleepers could have showed other factors, beyond age and BMI, contributing for lower scores of sleep quality in comparison with good sleepers.

This study has limitations. First, this cross-sectional design does not allow causative inferences, and reversal causality (e.g., poor sleep as a consequence of worse quality of life) cannot be ruled out. Second, the sample size is relatively low, which may have precluded finding significant associations in the regression models for some variables (e.g., handgrip). Third, we were unable to objectively assess sleep quality and sleep disorders (e.g., insomnia, central sleep apnea and narcolepsy). Forth, we did not assess controls (e.g., older without obesity or younger with obesity) to distinguish the separate impact of aging and obesity on the associations.

In conclusion, poor sleep quality associates with lower appendicular lean mass and handgrip strength, higher body fat and scores of anxiety and depression, and poorer health-related quality of life in older individuals with obesity. Additionally, sleep quality was shown to be an independent predictor of ALM, health-related quality of life, anxiety and depression. These findings reveal sleep quality as an important risk factor for overall health status in older individuals with obesity, thus warranting further clinical studies to test potential strategies to improve sleep quality as a measure to prevent poor outcomes in this understudied population.

## Methods

### Study design and participants

This is a cross-sectional study conducted in Sao Paulo (Brazil) between June 2021 and July 2022. Older individuals were recruited from the Division of Geriatrics of the School of Medicine of the University of Sao Paulo, and through advertisements on social media and older persons care centers. Men and women aged ≥ 65 years with current obesity (i.e.; body mass index [BMI] ≥ 30 kg/m^2^) were eligible to participate in the study. Exclusion criteria were as follows: medical conditions precluding physical testing and/or affecting the ability to complete questionnaires, no previous diagnose of sleep disorders and no history of cancer in the last 5 years.

The study was approved by the Ethics Committee of the Clinical Hospital of the School of Medicine of the University of São Paulo (04234918.1.0000.0065) and all procedures were in accordance with the recommendations of the Helsinki Declaration. The participants provided written informed consent before entering the study.

### Sleep quality assessment

Sleep quality was evaluated using the Portuguese version of Pittsburgh Sleep Quality Index (PSQI). In brief, the PSQI assesses sleep quality over a 1-month period by means of a questionnaire involving 19 self-rated questions and 5 questions answered by bedmates/roommates. The latter questions are used only for clinical information, and thus, were not used in the current study. The 19 questions are categorized into 7 components, graded on a score that ranges from 0 to 3. The PSQI components are as follows: subjective sleep quality (C1), sleep latency (C2), sleep duration (C3), habitual sleep efficiency (C4), sleep disturbances (C5), use of sleeping medication (C6) and daytime dysfunction (C7). The sum of scores for these 7 components yields one global score, which ranges from 0 to 21, in which the highest score indicates worst sleep quality. A global PSQI score greater than 5 indicates major difficulties in at least 2 components or moderate difficulties in more than 3 components^36^. Participants were classified as good sleepers in case of PSQI ≤ 5 and poor sleepers for PSQI > 5 (the higher the PSQI, the worse the sleep quality).

### Body composition and handgrip strength assessment

Body weight was assessed on a calibrated digital scale and height was evaluated with the aid of a stadiometer, from which BMI was calculated. All participants underwent a whole‐body dual‐energy x‐ray absorptiometry scan (DXA; Hologic QDR4500®, Hologic, Inc., Bedford, MA, USA) to quantify fat and lean mass, using Hologic APEX™ software. Appendicular lean mass (ALM) was calculated by summing the lean mass of all four limbs. All DXA measurements were carried out by the same trained technician.

Handgrip strength assessments were performed on both hands with the patient seated, shoulder adducted and neutrally rotated, holding the dynamometer (Jamar®; Sammons Preston Rolyan, USA) with the elbow positioned at a 90° angle. Three maximum attempts of 5 s on each hand with 1 min of the interval between attempts were performed, and the best result was used for analysis.

### Health related quality of life, anxiety and depression assessment

Health related quality of life was assessed using the short form (36) health survey (SF-36) questionnaire. The SF-36 questionnaire is self-administered and contains 36 items divided into eight domains: functional capacity, physical aspects, pain, general health, vitality, social aspects, emotional aspects, and mental health. It provides a score from 0 to 100, with lower scores indicating worse condition^37^.

Anxiety and depression were assessed using the Geriatric Anxiety Inventory (GAI)^38^ and the short form of Geriatric Depression Scale (GDS) with 15 items (GDS-15)^39^, respectively.

### Statistical analysis

Data are presented as absolute (n) and relative (%) frequency, means ± SD. Data normality was determined via Shapiro–Wilk test and visually checked with histograms. As all data presented normal distribution, independent *t-tests* were performed to test possible between-group differences (good sleepers vs. poor sleepers) for all dependent variables.

**Figure 3:**
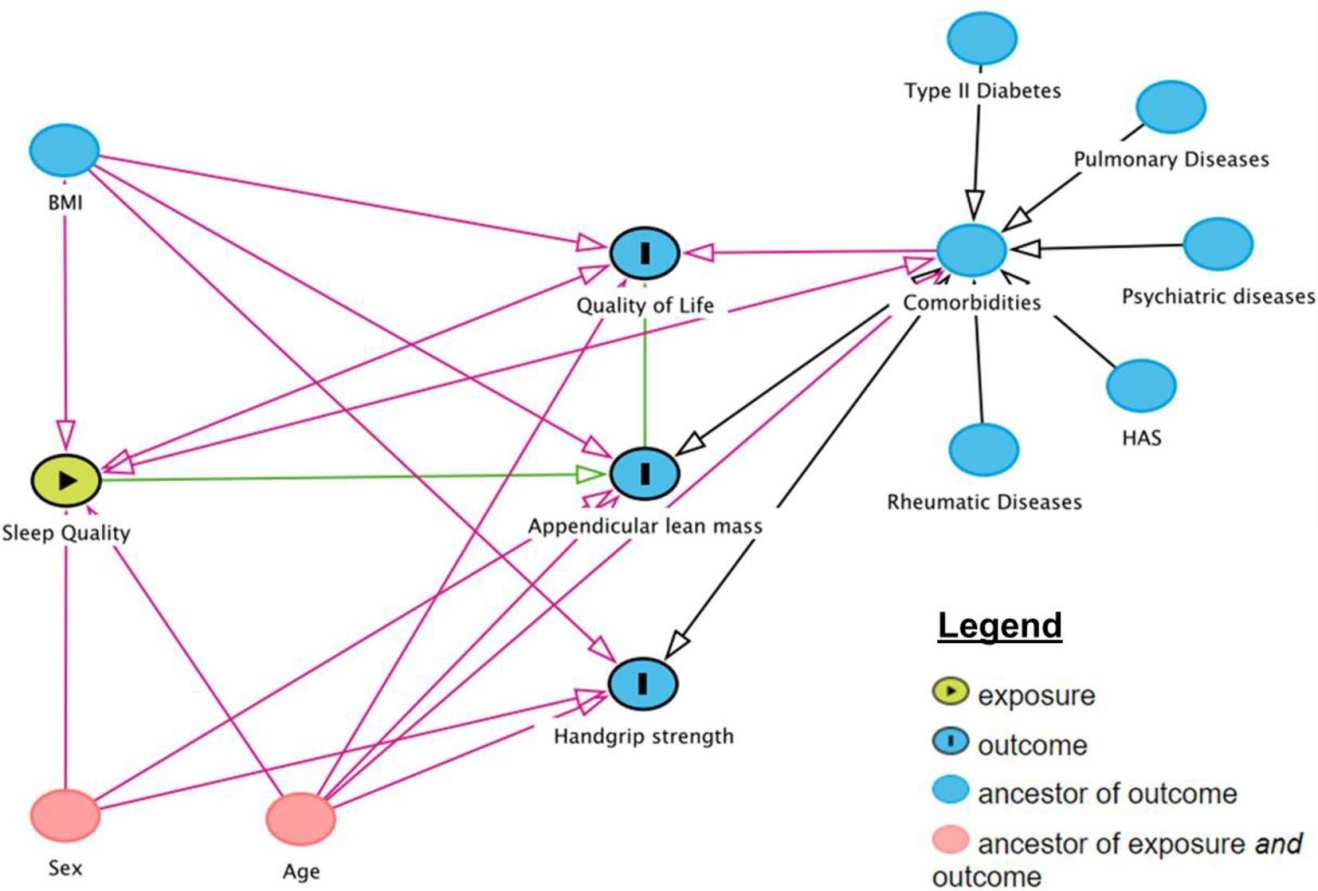
Direct acyclic graph of the association between sleep quality and appendicular lean mass (ALM), handgrip strength and quality of life. BMI: body mass index. Data is presented as individual data, mean, and standard deviation. *Indicates P < 0.05 for between-group comparisons using independent t-test.

Crude and adjusted linear regression models were utilized for verify possible associations between sleep quality and outcomes of interest (i.e., ALM, handgrip strength, and health related quality of life scores). Adjusted linear regression models are presented as follow: model 1: adjusted by age (as continuous variable) and sex (male or female). This model was based on a Direct Acyclic Graph (DAG, www.dagitty.net), which is a causal diagram based on causal relations between the exposure, outcome, and potential confounders (Fig. 3); model 2: adjusted by age (as continuous variable), sex (male or female), body mass index (as continuous variable), type II diabetes (yes or no), pulmonary diseases (yes or no), psychiatric diseases (yes or no), hypertension (yes or no) and rheumatic disease (yes or no). Beta coefficients were calculated along their corresponding 95% confidence intervals (95% CI). Significance level was set at *p* ≤ 0.05. All analyses were performed in the statistical environment R (version 3.5.3; R Core Team 2020).

## Supplementary Information

Supplementary Information.

## Data Availability

All background information on individuals and clinical information for patients included in this study are available from corresponding author on reasonable request.
