# Supplementary material for: Sleep quality is a predictor of muscle mass, strength, quality of life, anxiety and depression in older adults with obesity
Source: Sci Rep. 2023 Jul 12;13:11256. doi: 10.1038/s41598-023-37921-4 (PMC10338524; doi:10.1038/s41598-023-37921-4)
Supplement: Supplementary file 1 — Supplementary Information. [file 41598_2023_37921_MOESM1_ESM.docx]

**SUPLEMENTARY FIGURES**

**
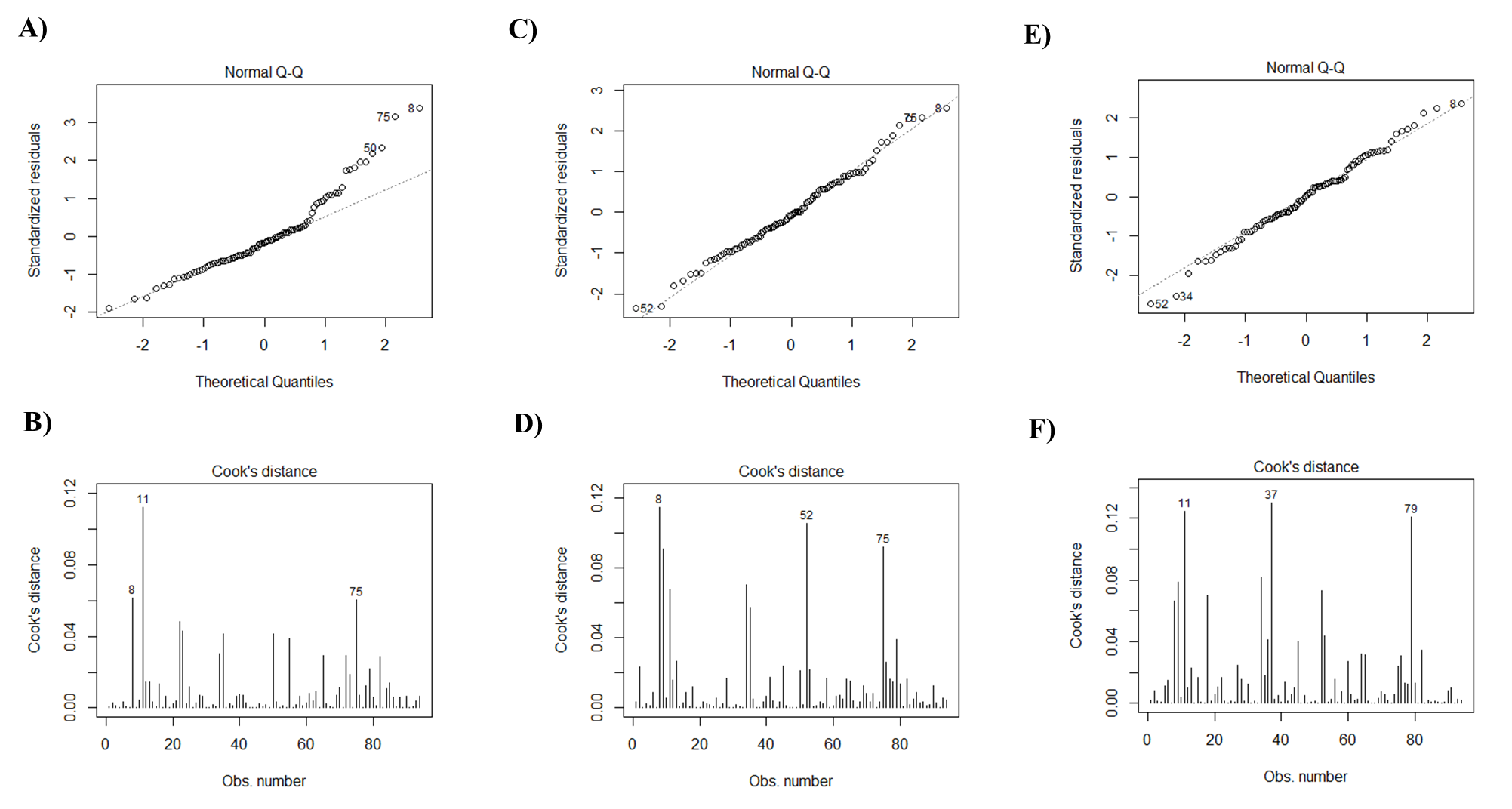
Figure S1.** Q-Q plot and Cook’s distance of model 1 (Panels A and B), 2 (Panels C and D) and 3 (Panels E and F) for prediction of appendicular lean mass.

**
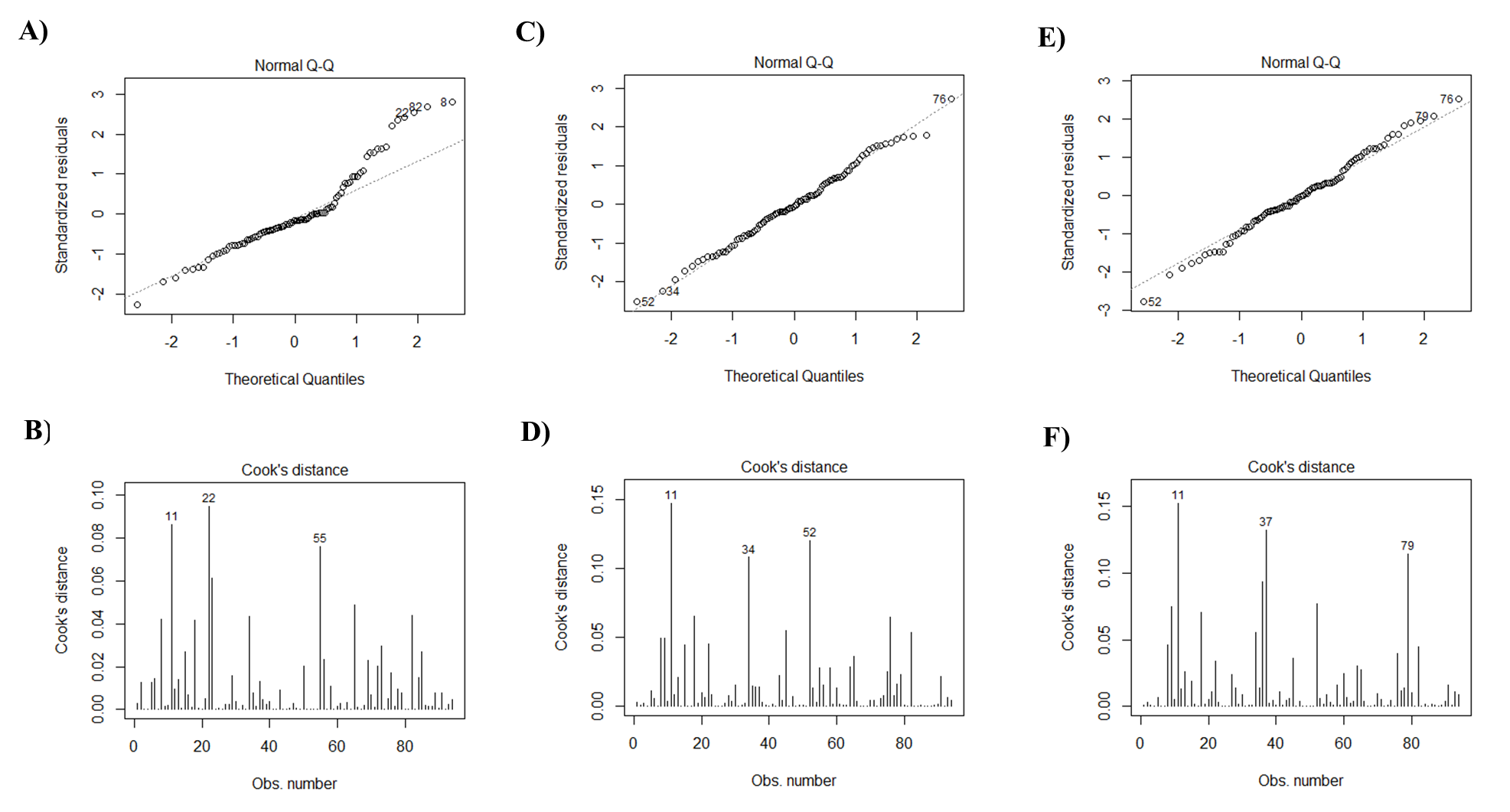
Figure S2.** Q-Q plot and Cook’s distance of model 1 (Panels A and B), 2 (Panels C and D) and 3 (Panels E and F) for prediction of appendicular lean mass adjusted by body mass index.


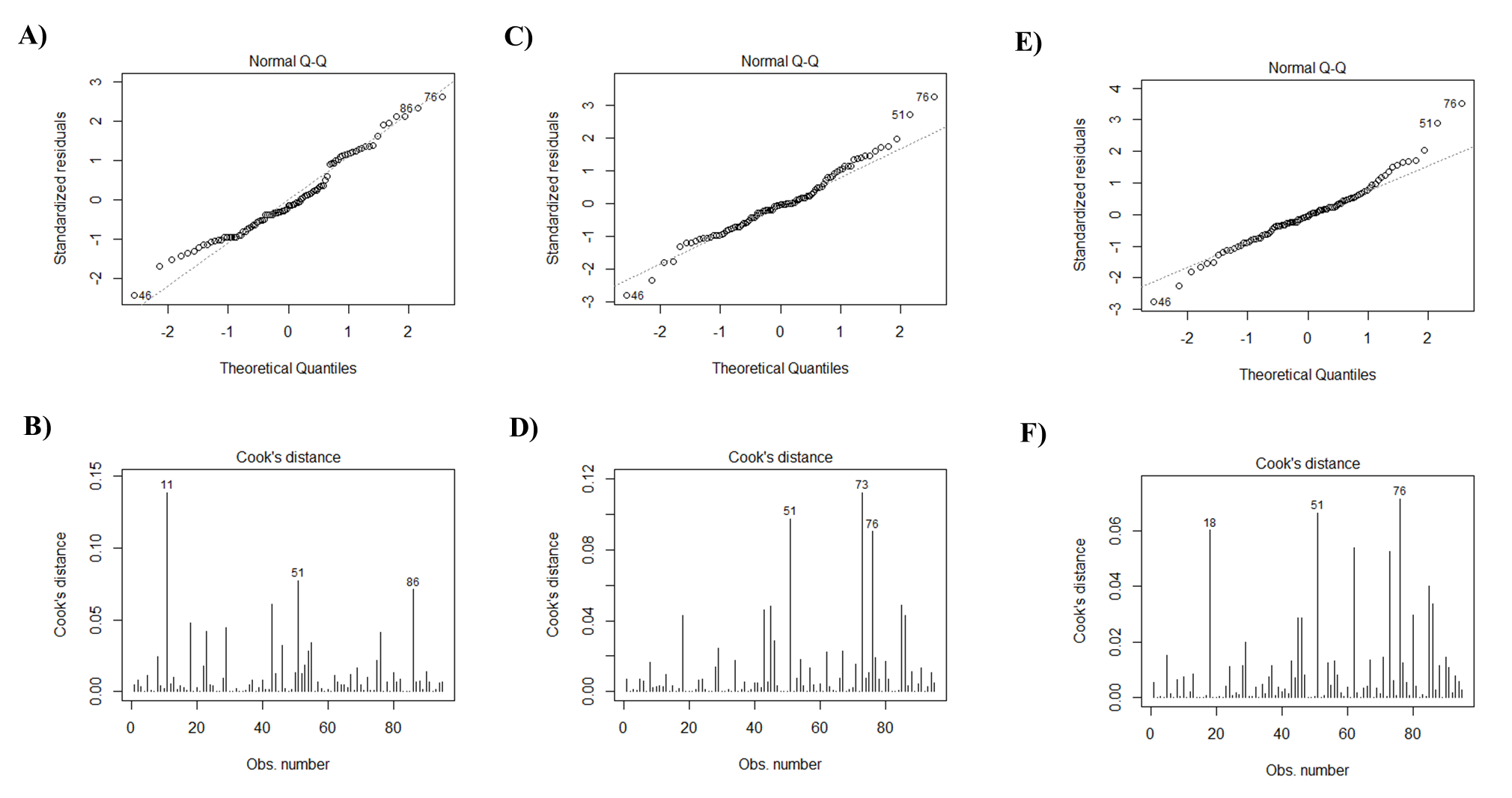
 **Figure S3.** Q-Q plot and Cook’s distance of model 1 (Panels A and B), 2 (Panels C and D) and 3 (Panels E and F) for prediction of handgrip strength.

**
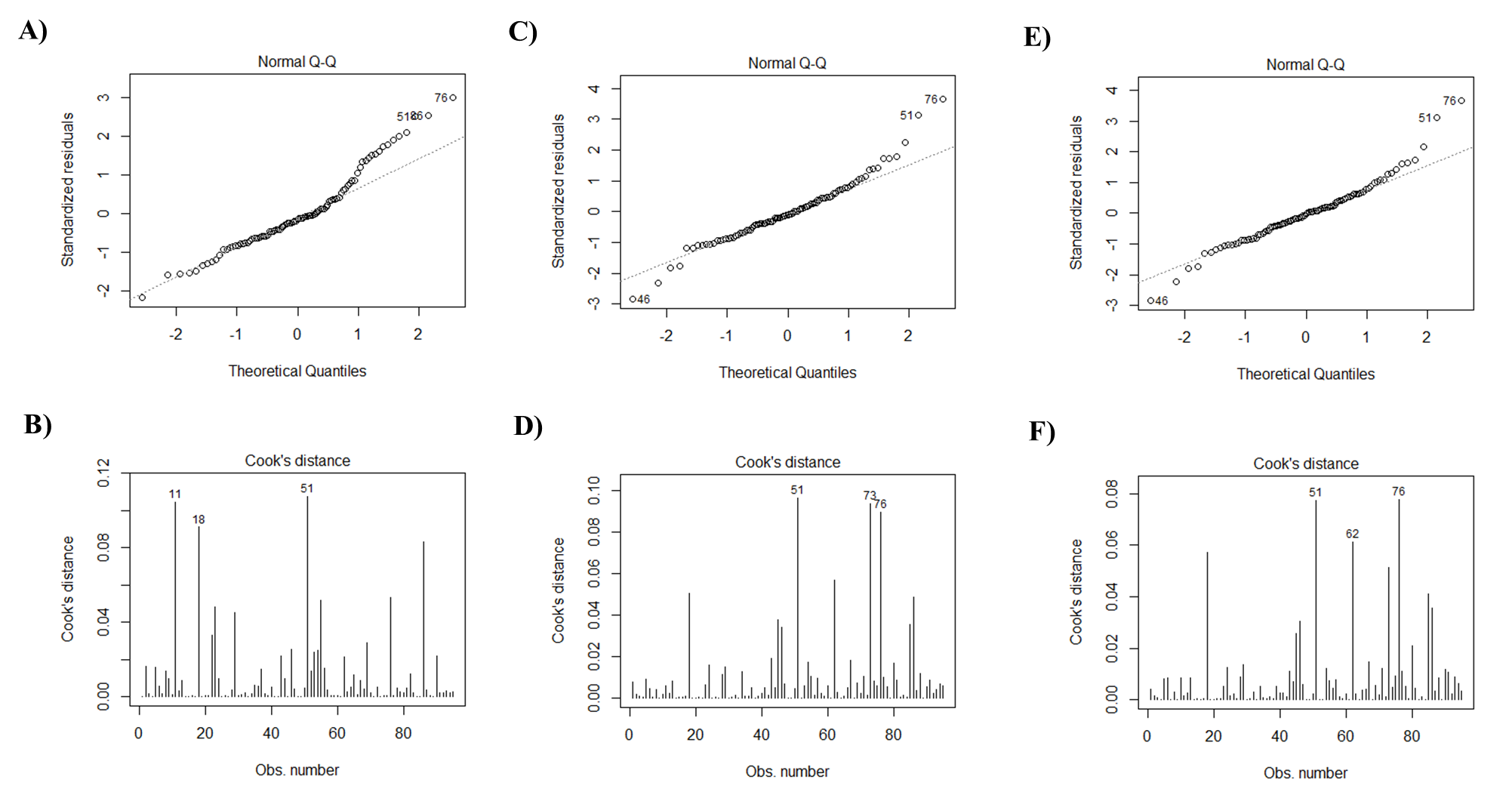
**

**Figure S4.** Q-Q plot and Cook’s distance of model 1 (Panels A and B), 2 (Panels C and D) and 3 (Panels E and F) for prediction of handgrip strength adjusted by body mass index.

**
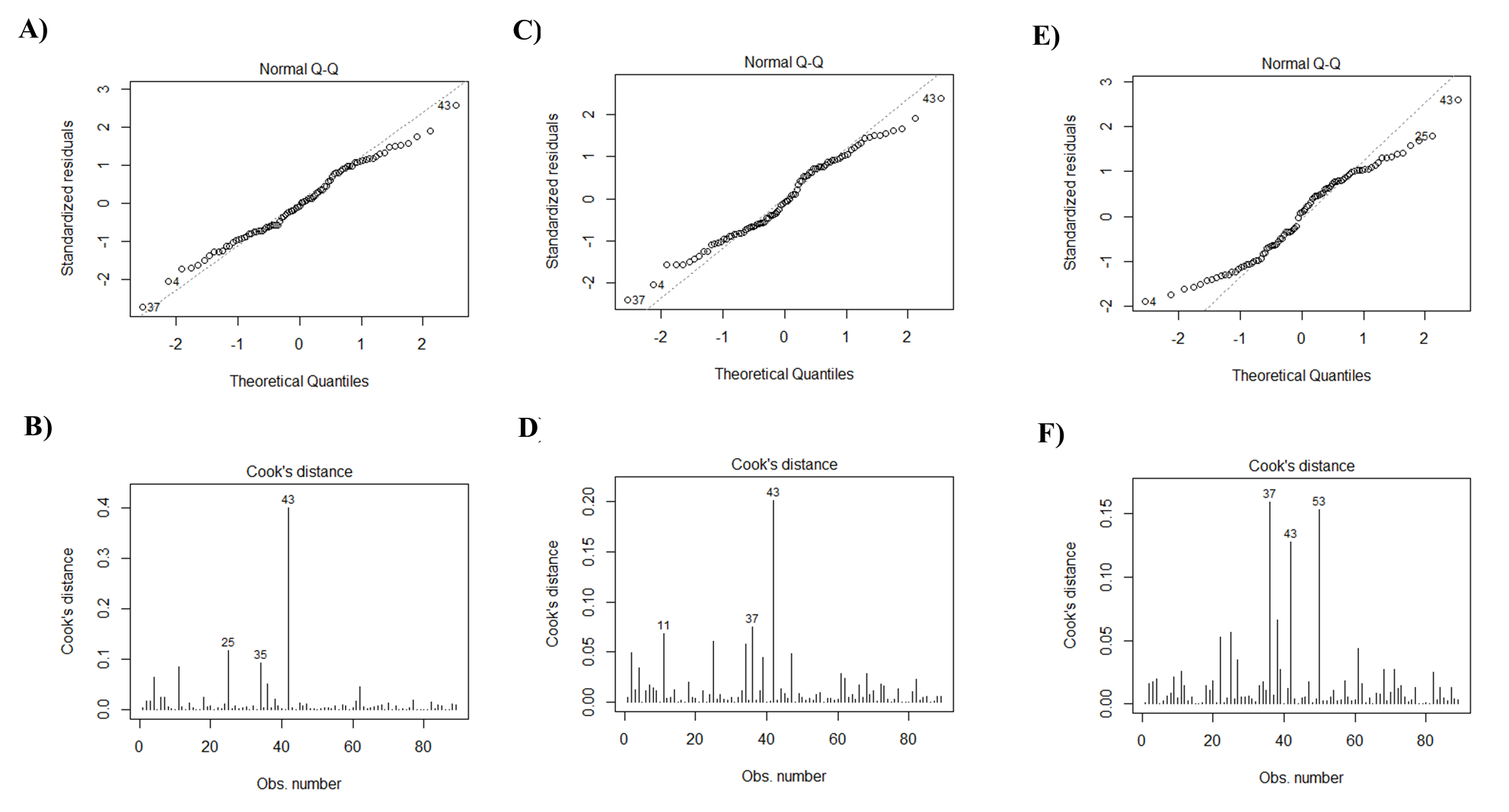
**

**Figure S5.** Q-Q plot and Cook’s distance of model 1 (Panels A and B), 2 (Panels C and D) and 3 (Panels E and F) for prediction of quality of life (physical health domain).

**
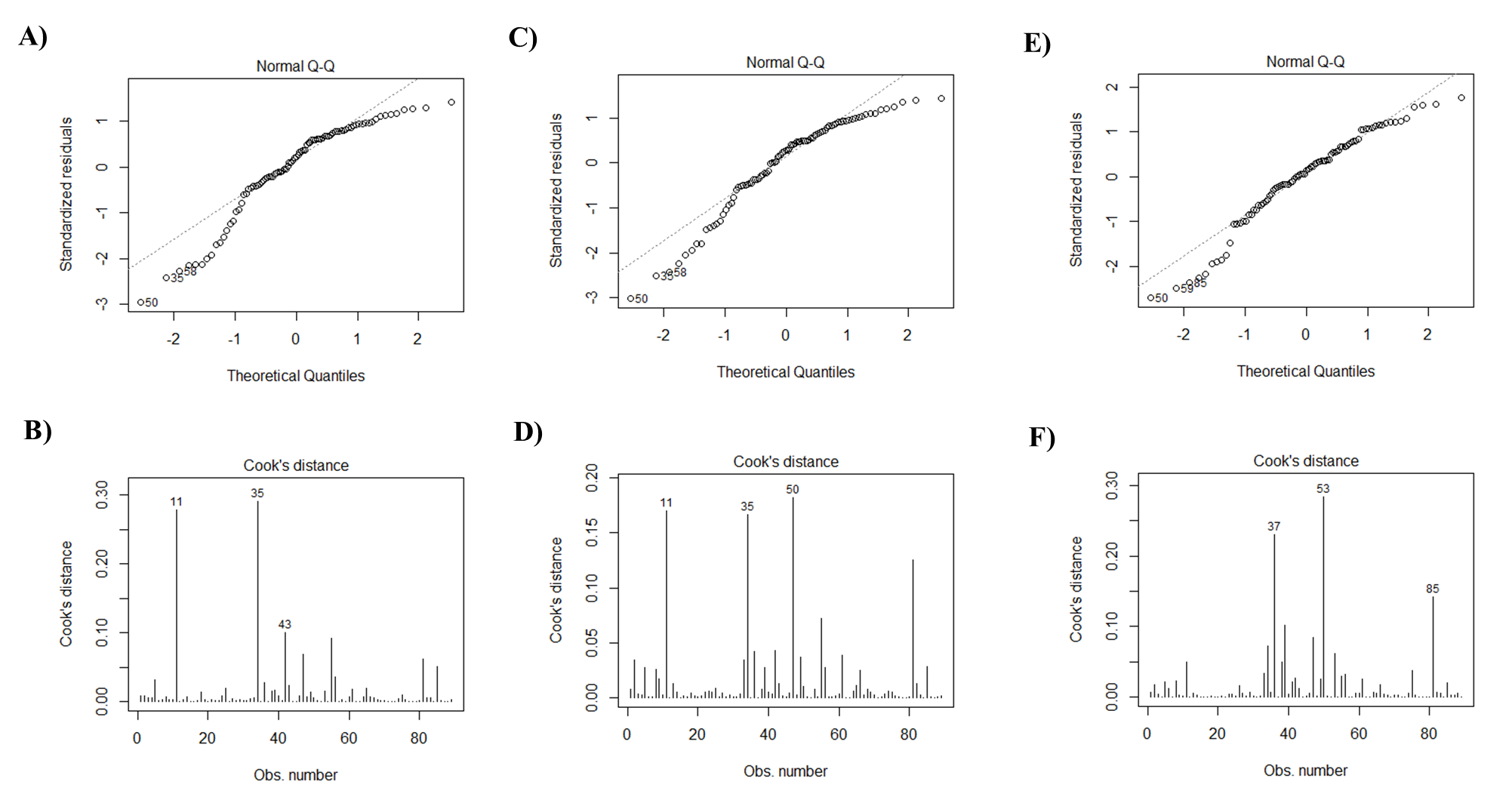
**

**Figure S6.** Q-Q plot and Cook’s distance of model 1 (Panels A and B), 2 (Panels C and D) and 3 (Panels E and F) for prediction of quality of life (mental health domain).

**
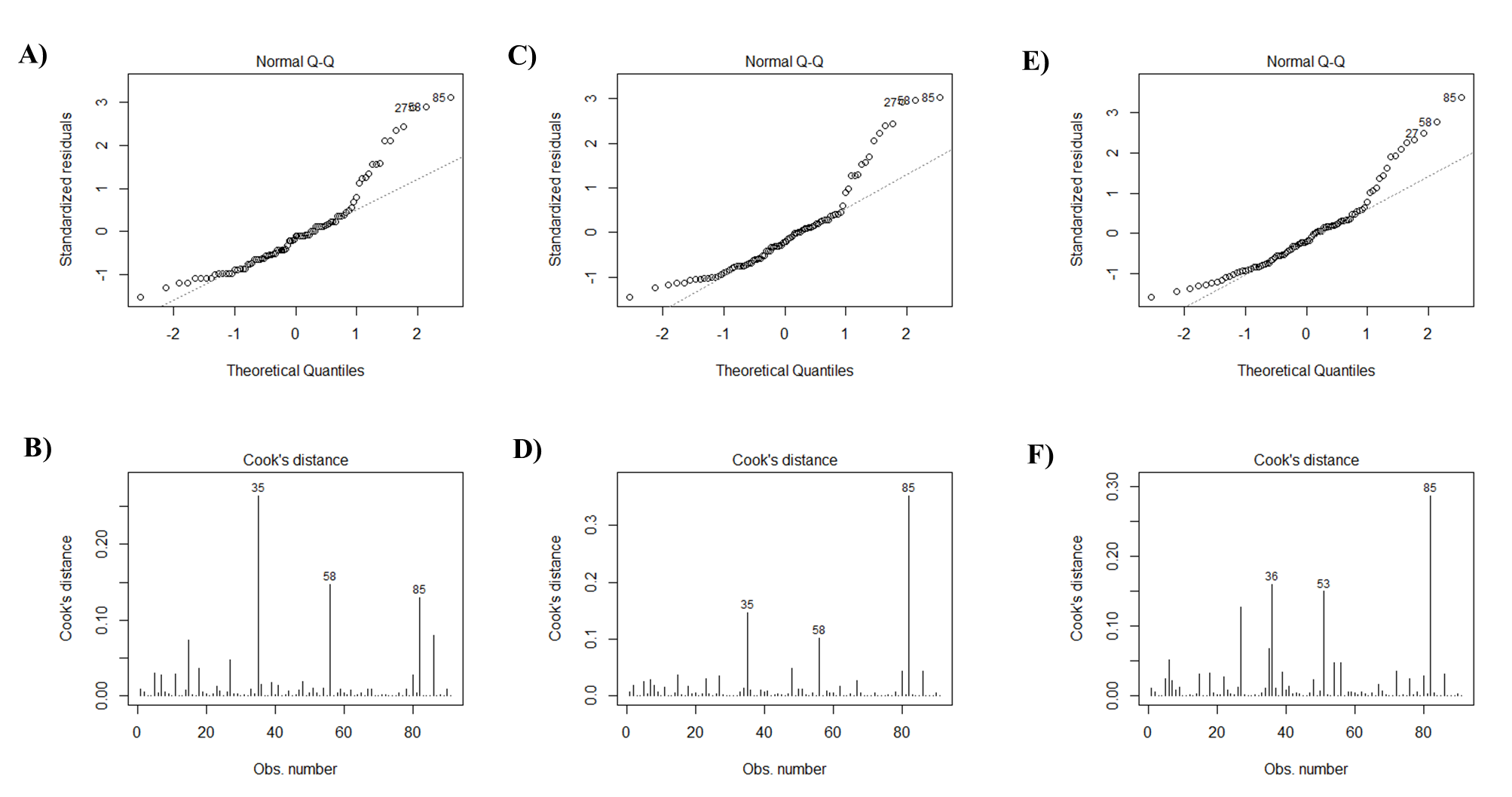
**

**Figure S7.** Q-Q plot and Cook’s distance of model 1 (Panels A and B), 2 (Panels C and D) and 3 (Panels E and F) for prediction of scores of anxiety.

**
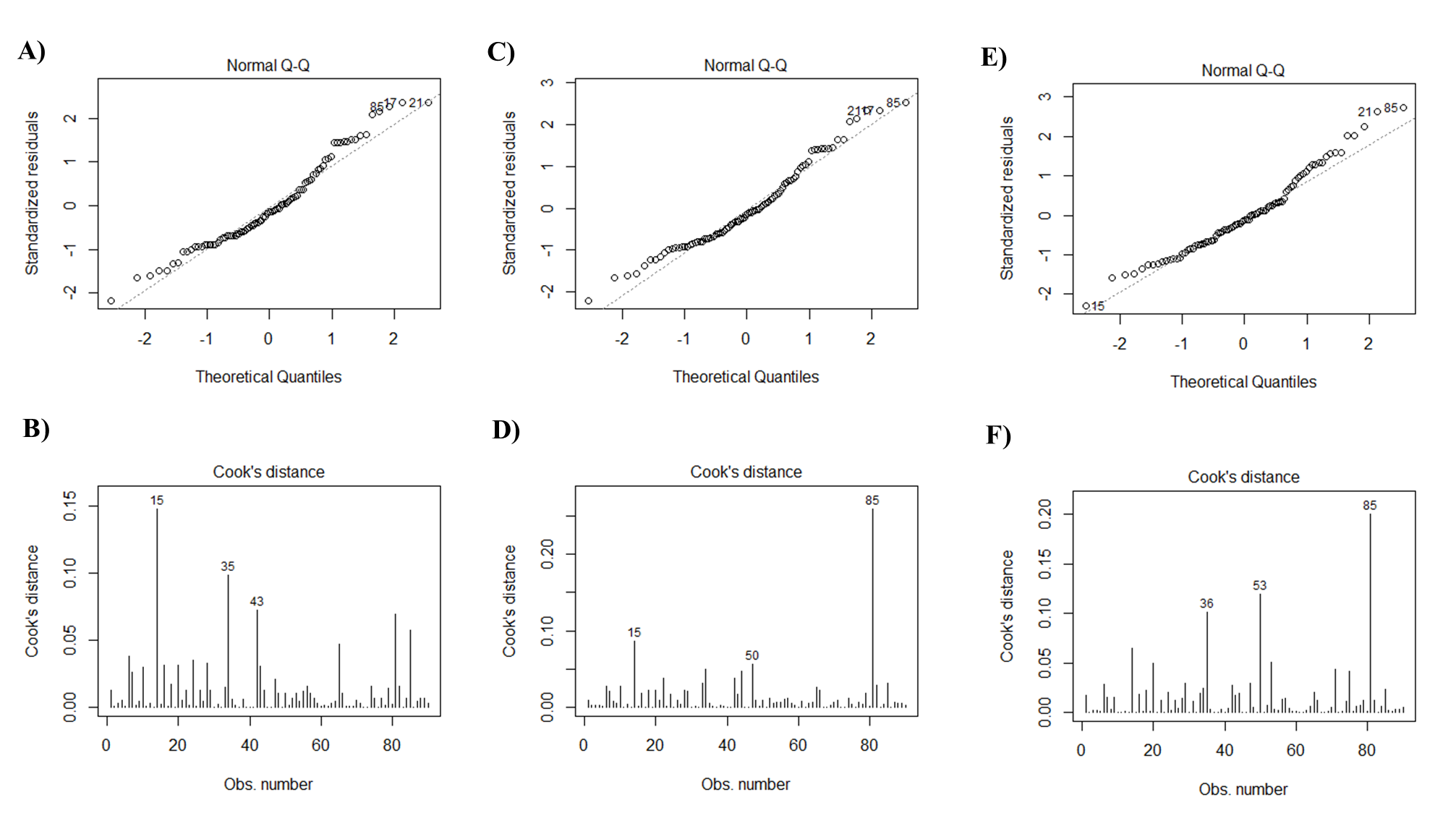
**

**Figure S8.** Q-Q plot and Cook’s distance of model 1 (Panels A and B), 2 (Panels C and D) and 3 (Panels E and F) for prediction of scores of depression.

**SUPLEMENTARY TABLES**

| **Table S1.** Correlation coefficient (R^2^) and Root mean square error (RMSE) of each model for all dependent variables. | | | | | | |
| --- | --- | --- | --- | --- | --- | --- |
|  | **Crude model** | | **Model 1 ^a^** | | **Model 2 ^b^** | |
| **Outcome** | **R^2^** | **RMSE** | **R^2^** | **RMSE** | **R^2^** | **RMSE** |
| ALM | 0.021 | 3.358 | 0.521 | 2.349 | 0.657 | 1.988 |
| ALM/BMI | 0.033 | 0.099 | 0.630 | 0.061 | 0.667 | 0.058 |
| Handgrip strength | 0.020 | 5.653 | 0.378 | 4.506 | 0.427 | 4.325 |
| Handgrip strength/BMI | 0.029 | 0.179 | 0.449 | 0.135 | 0.460 | 0.133 |
| SF-36 – Physical domain | 0.218 | 19.044 | 0.257 | 18.560 | 0.385 | 16.885 |
| SF-36 – Mental domain | 0.169 | 19.236 | 0.189 | 19.001 | 0.270 | 18.023 |
| Geriatric Anxiety Inventory | 0.163 | 5.070 | 0.166 | 5.062 | 0.226 | 4.874 |
| Geriatric Depression Scale | 0.134 | 3.012 | 0.140 | 3.002 | 0.199 | 2.898 |
| ALM: appendicular lean mass; BMI: body mass index; SF-36: Short form (36) health survey.  a = Linear regression models were adjusted by age (as continuous variable) and sex (male or female).  b = Linear regression models were adjusted by age (as continuous variable), sex (male or female), body mass index (as continuous variable), type II diabetes (yes or no), pulmonary diseases (yes or no), psychiatric diseases (yes or no), hypertension (yes or no) and rheumatic disease (yes or no). | | | | | | |

| **Table S2.** Generalized variance inflation factor for each predictor and model for all dependent variables. | | | | | | | | | | |
| --- | --- | --- | --- | --- | --- | --- | --- | --- | --- | --- |
|  |  | Sleep quality | Age | Sex | BMI | Type II diabetes | Pulmonary diseases | Hypertension | Psychiatric disease | Rheumatic diseases |
| ALM | Model 2 ^a^ | 1.003 | 1.005 | 1.003 | - | - | - | - | - |  |
|  | Model 3 ^b^ | 1.053 | 1.160 | 1.066 | 1.219 | 1.124 | 1.167 | 1.130 | 1.117 | 1.056 |
| ALM/BMI | Model 2 ^a^ | 1.003 | 1.005 | 1.003 | - | - | - | - | - |  |
|  | Model 3 ^b^ | 1.053 | 1.152 | 1.059 | 1.189 | 1.119 | 1.168 | 1.102 | 1.117 | 1.060 |
| Handgrip strength | Model 2 ^a^ | 1.005 | 1.003 | 1.004 | - | - | - | - | - |  |
|  | Model 3 ^b^ | 1.054 | 1.158 | 1.058 | 1.186 | 1.121 | 1.168 | 1.092 | 1.118 | 1.055 |
| Handgrip strength/BMI | Model 2 ^a^ | 1.003 | 1.003 | 1.004 | - | - | - | - | - |  |
|  | Model 3 ^b^ | 1.054 | 1.158 | 1.058 | 1.186 | 1.121 | 1.168 | 1.092 | 1.118 | 1.055 |
| SF-36 – Physical domain | Model 2 ^a^ | 1.001 | 1.003 | 1.004 | - | - | - | - | - |  |
|  | Model 3 ^b^ | 1.051 | 1.161 | 1.065 | 1.204 | 1.123 | 1.170 | 1.113 | 1.124 | 1.045 |
| SF-36 – Mental domain | Model 2 ^a^ | 1.051 | 1.003 | 1.004 | - | - | - | - | - |  |
|  | Model 3 ^b^ | 1.051 | 1.161 | 1.065 | 1.204 | 1.123 | 1.170 | 1.113 | 1.124 | 1.045 |
| Geriatric Anxiety Inventory | Model 2 ^a^ | 1.001 | 1.004 | 1.004 | - | - | - | - | - |  |
|  | Model 3 ^b^ | 1.055 | 1.056 | 1.162 | 1.129 | 1.122 | 1.167 | 1.109 | 1.116 | 1.073 |
| Geriatric Depression Scale | Model 2 ^a^ | 1.001 | 1.003 | 1.004 | - | - | - | - | - |  |
|  | Model 3 ^b^ | 1.0.53 | 1.160 | 1.066 | 1.219 | 1.124 | 1.167 | 1.110 | 1.117 | 1.056 |
| ALM: appendicular lean mass; BMI: body mass index; SF-36: Short form (36) health survey.  a = Linear regression models were adjusted by age (as continuous variable) and sex (male or female).  b = Linear regression models were adjusted by age (as continuous variable), sex (male or female), body mass index (as continuous variable), type II diabetes (yes or no), pulmonary diseases (yes or no), psychiatric diseases (yes or no), hypertension (yes or no) and rheumatic disease (yes or no). | | | | | | | | | | |
